# Safety and efficacy of radiation and chemoradiation in patients over 70 years old with inoperable esophageal squamous cell carcinoma

**DOI:** 10.3892/ol.2013.1694

**Published:** 2013-11-20

**Authors:** HONG-YU XU, ZE-DONG DU, LIN ZHOU, MIN YU, ZHEN-YU DING, YOU LU

**Affiliations:** 1 Department of Thoracic Oncology and State Key Laboratory of Biotherapy, West China Hospital, West China Medical School, Sichuan University, Sichuan 610041, P.R. China; 2 Oncology Department, 363 Hospital, Chengdu, Sichuan 610041, P.R. China

**Keywords:** esophageal squamous cancer, elderly, chemoradiation, radiation, toxicity

## Abstract

The aim of the present study was to perform a retrospective analysis to investigate the outcome and toxicity of radiation (RT) and chemoradiation (CRT) in elderly, inoperable patients >70 years old. Between 2003 and 2012, 1,024 patients with squamous cell carcinoma (SCC) of the esophagus were treated at the Department of Thoracic Cancer, West China Hospital (Chengdu, China). Of these patients, 37 were >70 years old and had not undergone surgery, and were selected for analysis. Of these 37 patients, CRT had been administered to 20 (54%). Actuarial survival rates were determined by the Kaplan-Meier method. The one-year survival rate in the CRT group (n=20) was 85%, while 35% of patients in the RT group (n=17) survived for more than one year. The overall and progression-free survival in the CRT group versus the RT group were 17 months [95% confidence interval (CI), 11.861–22.139] versus eight months (95% CI, 6.674–9.326) (P=0.013) and 14 months (95% CI, 9.617–18.383) versus five months (95% CI, 2.311–7.689) (P=0.01), respectively. Patients irradiated with a dose of >50 Gy exhibited an improved survival rate compared with patients who received a dose of ≤50 Gy (18 vs. 14 months; P=0.049). Furthermore, patients with an Eastern Cooperative Oncology Group (ECOG) score of ≤1 had an improved prognosis compared with those with an ECOG score of 2 (14 vs. seven months; P=0.006). The two regimens were well-tolerated and there were no therapy-associated mortalities. The current retrospective study indicated that patients of >70 years old with inoperable esophageal SCC and a good ECOG score exhibit comparably better safety levels with CRT and improved survival rates compared with RT alone.

## Introduction

Esophageal cancer (EC) is the sixth leading cause of cancer mortality, with ~17,460 new cases (13,950 males and 3,510 females) and 15,070 mortalities reported in 2012 (1). At diagnosis, ~30% of EC patients are >70 years old. The overall five-year relative survival rate between 2002 and 2008 from 18 SEER geographical areas was 16.9%. In China, the morbidity of EC ranks fourth and is the fifth leading cause of cancer-related mortality (2). The major histological type in China is squamous cell carcinoma (SCC), which accounts for >90% of all types of EC (3,4). Therefore, the control of this disease is an urgent issue.

Esophagectomy is the preferred first-line treatment for patients with localized or regional EC. However, only an extremely small number of elderly EC patients receive surgical resection due to the high operative mortality associated with old age and specific cases where patients are regarded as medically unfit for surgery. Moreover, esophagectomy has not been shown to be superior to radiation (RT) alone, even in resectable cases (5,6). In practice, for stage I–IIIA SCC of the esophagus, chemoradiotherapy (CRT) is superior to RT alone, with longer survival durations and higher remission rates (7,8). Hence, according to the National Comprehensive Cancer Network (NCCN) Clinical Practice Guidelines in Oncology™, version.2.2012 (9), CRT or RT alone is proposed as the standard treatment for localized or regional EC patients who are medically unfit for surgery.

To the best of our knowledge, 35% of EC patients exhibit distant metastasis at diagnosis (1) and are not cured with multimodality therapy. However, palliative therapy is required to maintain the patient’s ability to swallow for the delivery of nutrition, prevent hemorrhage and relieve pain. RT and CRT are important for palliative therapy (10). A previous retrospective study showed that, following palliative CRT, dysphasia scores improved in 75% of the patients and 85% of patients improved their oral intake, no longer requiring support, in a median time of 43 days (11).

Although a number of studies have reported that CRT presents a great benefit to EC patients who are medically unfit for surgery and no severe side effects have been reported, few studies have focused on elderly patients. The majority of elderly patients with EC often have an increased number of comorbidities, lower performance status (PS) and are reluctant to undergo surgical practice with high risk (12). To date, no specific studies have identified standard therapeutic strategies for elderly patients with EC. Therefore, the current retrospective study was designed to evaluate the efficacy and toxicity of CRT or RT in elderly patients with EC to identify the best method of treatment.

## Patients and methods

### Patients

Between January 2003 and March 2012, 1,024 patients with SCC of the esophagus were treated by RT or CRT at the Department of Thoracic Oncology (West China Hospital, Chengdu, China), including 151 patients >70 years old. Of these patients, 43 had not undergone surgery. Overall, 37 patients were investigated following the exclusion of six patients due to incomplete medical records. Patients who met the following criteria were eligible: i) pathologically confirmed SCC of the esophagus; ii) complete and retrievable clinical records; iii) ≥70 years old; iv) ECOG PS of ≤2; v) patients or family members were contactable; vi) clear survival status; and vii) had not undergone surgical resection. The main reasons for inoperability were as follows: i) stage IV disease (13 patients); ii) medical issues (11 patients; five with cardiopathy, four with pulmonary disease and two with cerebra-vascular disorders); iii) advanced age alone (five patients); and iv) patient refusal (eight patients).

A predesigned form was used to record specific information, including age, gender, tumor diagnosis, stage, current treatment, survival status and toxicity. All patient information was carefully reviewed and accurately recorded. The study was approved by the ethics committee of West China Hospital.

### Treatment

#### RT

A three-dimensional (3D) plan was used for 12 patients, 19 patients were treated by intensity-modulated radiation therapy (IMRT), two patients received image guide radiation therapy (IGRT), three patients received volumetric modulated arc therapy (VMAT) and only one patient was treated with a two-dimensional plan. The clinical target volume (CTV) was defined as ≥5 cm proximally and distally and 1 cm laterally beyond the gross tumor volume (GTV), as delineated by a computed tomography (CT) scan and included adjacent lymph nodes. The RT therapy parameters were as follows: i) fractionation, 200 cGy each time; ii) GTV, primary tumor and macroscopically involved lymph nodes; iii) CTV, primary tumor and the area of subclinical involvement surrounding the GTV; iv) planning target volume, including a minimum of 0.5–1-cm surrounding the CTV; v) field size, based on the tumor size; and vi) energy, 6 or 8 MV. The various RT dosages were selected according to the clinical conditions of the patient. By conventional fractionation, patients were delivered 2 Gy per fraction, one fraction per day and five fractions per week.

#### Chemotherapy

Patients concomitantly received chemotherapy once a month for four cycles of PF [75 mg/m^2^ cisplatin on day 1 and 750 mg/m^2^ 5-fluorouracil (5-FU) daily for five consecutive days] or FO (130 mg/m^2^ oxaliplatin on day 1 and 750 mg/m^2^ 5-FU daily for five consecutive days) regimen or three weeks of TP regimen (135 mg/m^2^ paclitaxel and 75 mg/m^2^ cisplatin on day 1 or 130 mg/m^2^ oxaliplatin on day 1).

#### Evaluation of response and toxicity

Tumor response was evaluated by the Response Evaluation Criteria in Solid Tumors version 1.1 (13). Overall survival (OS) was calculated from the date of RT or CRT initiation up to the date of mortality or last follow-up. Progression-free survival (PFS) was calculated from the first dose of treatment to the first evidence of tumor progression or the date of the last follow-up. This evaluation was performed six to eight weeks following CRT or RT completion. The follow-up was performed on a clinical basis, with upper digestive endoscopy with biopsy and chest and abdominal CT scans every three months.

Follow-up data were updated in April 2013. Physician-reported acute hematological, esophageal and pulmonary toxicities of all eligible patients were evaluated according to the Radiation Therapy Oncology Group (RTOG) scales, while gastrointestinal reaction was scored by the National Cancer Institute Common Toxicity Criteria, version 3.0 (14).

#### Statistical methods

Tumor response, PFS and OS were analyzed. Survival curves were determined using the Kaplan-Meier method. Prognostic factors of survival were examined by univariate analysis to estimate the hazard ratio (HR) with 95% confidence interval (CI). Seven predefined variables for the univariate analysis were ECOG score, lymph node involvement, distant metastasis, tumor length, RT dose, discontinuation of RT and tumor location. Any variables reaching P=0.05 were introduced into a multivariate analysis. P<0.05 was considered to indicate a statistically significant difference. Statistical analyses were performed using SPSS 17.0 (SPSS, Inc., Chicago, IL, USA).

## Results

### Patient follow-up

Thirty-seven patients with SCC of the esophagus who were >70 years old and treated with RT or CRT between January 2003 and March 2012 were eligible for the present study. Individuals were followed up until April 2013. All 37 patients were able to be evaluated for toxicity and tolerability, and with the exception of two patients, the rest of the patients were evaluated for response. The median follow-up period was 64 weeks (range, 16–324 weeks). At the termination of the follow-up period, 21 patients had experienced tumor progression and 27 patients had succumbed to their condition. All individuals had exhibited improved symptoms of dysphasia.

### Patient characteristics

Patient pretreatment characteristics are listed in Table I. The median age of the patients was 76 years old (range, 70–88 years old). The median ECOG score was 1 (range, 0–2) with the majority of patients exhibiting a score of 0–1 (89.2%). Prior to treatment, the majority of patients had a good nutritional status with a median BMI of 20.7 (range, 14–35), albumin of 39.5 μmol/l (range, 29–47.7 μmol/l), good renal function with a median eGFR of 53.5 ml/min (range, 39.4–74.1 ml/min) and good liver function with median ALT of 15.6 IU/l (range, 4–34 IU/l) and median AST of 21.3 IU/l (range, 12–62 IU/l). Of the patients, 23 had a history of smoking and common comorbidities are listed in Table I.

### Tumor characteristics

All patients were examined by CT and barium esophagography. Esophagogastroduodenoscopy was not used routinely and the majority of patients refused this procedure due to invasiveness. Therefore, it was difficult to classify T stage by CT scan only. The majority of patients exhibited the involvement of at least one lymph node (48.6%) and 13 patients (54.1%) exhibited clinical evidence of distant metastasis (Table II). Primary metastasis was most commonly located in the upper and middle esophagus (67.5%) and 56.7% of patients exhibited a tumor length of 3–5 cm.

### Treatment characteristics

CRT and RT treatment characteristics are listed in Table III. Twenty patients received definitive CRT, including twelve patients who had concurrent CRT. All patients received 3D conformal RT, with the exception of one patient treated by 2D-RT, including 19 IMRT, two IGRT and three VMAT. The median delivered dose of RT was 51.5 Gy (range, 36–66 Gy). In addition, 21 patients received RT doses of >50 Gy and 16 patients received RT doses of ≤50 Gy. Discontinued RT was reported in 27% of patients due to intolerance to acute RT reactions and seven patients had RT intervals of greater than one week. Chemotherapy was prescribed for 20 patients and the most commonly used chemotherapy regimen was 5-FU and cisplatin (35%). The majority of patients received platinum agents (90%) with the exception of two patients who received paclitaxel and xeloda, respectively. Concurrent chemotherapy was administered in 12 cases and the remaining patients were prescribed chemotherapy following RT.

### Treatment response

All patients, with the exception of two, were evaluated for tumor response. Six patients achieved complete remission (CR; 17.1%) and six patients exhibited partial remission (PR; 17.1%). The objective response rate was 34.2%. In the CRT group, 40% of patients exhibited tumor remission and the disease control rate was 55%. However, in the RT group, the objective remission rate declined to 17.6% and only two patients exhibited stable disease. The differences in response rate between the two groups was of statistical significance (P=0.04).

Patients in the CRT group achieved improved tumor control rates (55 vs. 29.4%), but the difference was not statistically significant (P=0.057).

### Survival

**Figure 1:**
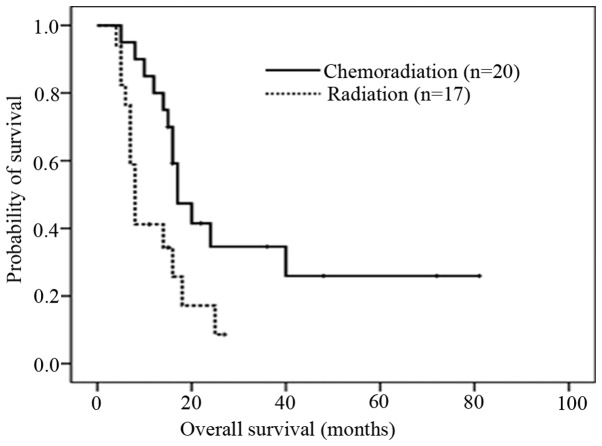
Kaplan-Meier curves for overall survival.

**Figure 2:**
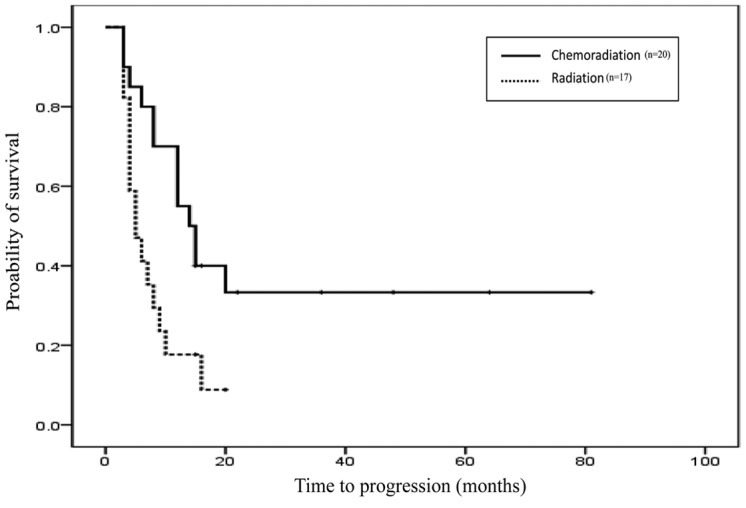
Kaplan-Meier curves for time to progression.

Disease progression was detected in 21 patients. The median PFS was nine months for all patients. In the CRT group the median PFS was 14 months (95% CI, 9.617–18.383) which was improved compared with the RT group with a median PFS of five months (95% CI, 2.311–7.689) (Fig. 1). The difference in PFS between the two groups was found to be statistically significant (P=0.01). The one-year PFS rate for the CRT group (n=20) was 50%, while 35% of patients in the RT group (n=17) did not exhibit tumor progression for more than one year. The median follow-up period was 64 weeks. When the final evaluation was performed, 27 patients had succumbed to their condition. The median OS time was 16 months for all patients. In the CRT group, the median OS was 17 months (95% CI, 11.861–22.139); whilst in the RT group, the median OS was just eight months (95% CI, 6.674–9.326). The difference in OS between the two groups was found to be statistically significant (P=0.013; Fig. 2). The one-year survival rate for the CRT group (n=20) was 85%, while 35% of patients in the RT group (n=17) survived for more than one year. For stage IV EC patients, six patients (75%) survived for >16 months in the CRT group, while all patients in the RT group failed to reach the median OS.

### Toxicity

Acute toxicity grades 3 and 4 were observed in 37.8 and 2.7% of patients, respectively (Table IV). Only one patient suffered from acute grade 4 neutropenia and thrombocytopenia in the CRT group. No patients experienced neutropenic fever during treatment. Acute grade 3–4 esophagitis was identified in 5.4% of the CRT patients and 2.7% of the RT patients. In addition, 5.4% of the CRT patients and 2.7% of the RT patients suffered from grade 3–4 pneumonitis. The majority of patients who had poor tolerability continued their RT plan following treatment for dysphasia, shortness of breath or even dyspnea with glucocorticoid. Only three patients refused to complete their plan due to severe side effects. Grade 3 gastrointestinal reactions were identified in 8.1% of the CRT group, but patients were able to tolerate treatment plans to completion with appropriate treatment for side effects.

### Univariate analysis

By univariate analysis, patients with good ECOG (PS, ≤1) were found to achieve higher survival rates compared with patients with a PS of 2. The difference between the two groups was statistically significant (P=0.006). Patients receiving ≥50 Gy RT exhibited improved survival rates compared with patients receiving ≤50 Gy and the difference was statistically significant (P=0.049). The discontinuation of RT and the length of RT intervals are likely to have no effect on OS (P=0.130 and 0.591, respectively). Multivariate analyses were not performed due to the small cohort size.

## Discussion

In patients with SCC of the esophagus, ~30% are >70 years old (1). However, few studies have focused on patients of this age group (Table V) (15,16). CRT is the standard treatment for individuals unfit for surgery and is superior to RT (8,9). A phase III, prospective, randomized and stratified trial (17) was performed to compare the CRT regimen of fluorouracil and cisplatin with RT alone. The median survival was 12.5 versus 8.9 months. The RTOG 85-01 trial (18) has shown a large difference in the five-year survival rates between CRT and RT groups (26 vs. 0%, respectively). However, the two trials have not focused on elderly patients. To the best of our knowledge, no studies have compared CRT with RT in elderly EC patients. Patients in the present study had a median age of 76 years-old (range, 70–88 years-old) and it was shown that CRT improves OS and PFS by nine months (17 vs. eight months; P=0.013; and 14 vs. five months; P=0.01, respectively) In the current study, there were certain patients with longer survival times, with one patient who survived for 73 months. Furthermore, with the exception of one patient, all patients received 3D-RT therapy, and we hypothesize that this was responsible for the higher median survival time compared with other previous studies (Table V) (19,20). The results of the present study indicate that patients with good ECOG score and limited comorbidities are able to tolerate CRT to completion and achieve a longer OS time (21).

CRT must not be ignored in stage IVB EC as a palliative therapy for patients with good ECOG score and limited comorbidities. For advanced stage IV EC, CRT is important for relieving patients from symptoms of swallowing difficulties (11). A clinical trial performed in Japan (11), which focused on stage IVB EC patients with a median age of 64 years old, showed that CRT is likely to achieve a good response rate (55% of patients) and relieve the symptoms of dysphasia, improving the quality of life of the patients with good tolerance (<20% of patients exhibited grade 3–4 toxicity). Compared with the current study, among 13 patients of stage IV EC, eight patients received CRT and 62.5% of patients achieved CR or PR. Therefore, this indicates that chemotherapy is likely to increase the response rate and enhance the effect achieved by RT even in patients with distant metastasis.

With regard to acute toxicity, the majority of patients were able to tolerate RT, since only one patient exhibited grade IV neutropenia. In the CRT group, there was a greater incidence of grade III–IV toxicities, particularly hematological side effects. The number of patients in the CRT group suffering from gastrointestinal side effects were greater compared with patients in the RT group. The side effects were primarily initiated by chemotherapy. The RT schedule was discontinued by 10% of patients mainly due to unbearable side effects, including acute esophagitis. Of note, the frequency of esophagitis in the RT group was greater compared with that in the CRT group. Therefore, we hypothesize that chemotherapy does not increase side effects associated with RT but may improve the efficacy of RT. In the present study, the incidence of grades III–IV were usually lower when compared with other studies (17,22). This is due to the fact that all patients, with the exception of one individual, received 3D-RT, including IMRT (19 patients), IGRT (two patients) and VMAT (three patients). Advanced RT technology is likely to deliver treatment of higher efficacy and lower toxicity to patients.

The main reported predictive factors of response to OS were WHO performance status, nutritional status, treatment dose and TNM stage (23,24). In the current study, the predictive factors of OS by univariate analysis was WHO performance status and doses of RT. Aside from these two factors (25,26), no significant differences were found in the results of the univariate analysis, controversial to previous studies. This was primarily due to the small cohort size.

Limitations with regard to the generalisability of the results of the present study include the fact that it was a retrospective study, hence the evaluation of non-hematological toxicity was primarily dependent on patient medical records, and specific minor side effects (particularly of <grade 2 non-hematological toxicity) were not monitored carefully. Additionally, survival rate, with regard to specific stratification factors, demonstrated a difference between the two groups; however, no significant difference was identified due to small cohort size. Moreover, patients did not receive identical chemotherapeutic regimens and ~50% of patients received fluoropyrimidine-based chemotherapy, while taxane-based chemotherapeutic regimens were selected for the other 50% of patients. However, according to the NCCN Clinical Practice Guidelines in Oncology (version 2.2012), the two chemotherapy regimens are standard for concurrent CRT and yield specific bias to the results of the current study. Further, the present study included ~35% of advanced stage IV EC patients; therefore, objective response and disease control rates were lower compared with those of other studies that included only diseases of ≤stage III (27). Therefore, future large-scale prospective clinical trials, particularly for elderly patients, are required.

Although certain limitations were observed in this small, retrospective study, the present study may have important implications for the therapy of SCC in elderly EC patients. CRT was found to be effective and safe for SCC of the esophagus in elderly patients and treatment compliance was observed to be good. PFS was prolonged by this combined regimen and an improved OS was observed. All these observations must be confirmed in a larger, prospective and randomized clinical trial.

## Figures and Tables

**Table I:** Pretreatment patient characteristics (n=37).

Characteristic	Value
Gender, n (%)	
Male	32 (86.5)
Female	5 (13.5)
Age at diagnosis, years	76 (70–89)^a^
70–75, n (%)	18 (48.6)
76–80, n (%)	14 (37.8)
>80, n (%)	5 (13.5)
ECOG score, n (%)	
0	11 (29.7)
1	22 (59.5)
2	4 (10.8)
Median weight, kg (range)	56.6 (40–75)
Patients with weight loss >10%, n (%)	4 (11.8)
Median BMI, kg/m^2^ (range)	20.7 (16–26)
Median estimated glomerular filtration rate, ml/min/1.73 m^2^ (range)	53.5 (39.4–74.1)
Median albumin, μmol/l (range)	39.5 (29–47.7)
Median ALT, IU/l (range)	15.6 (4–34)
Median AST, IU/l (range)	21.3 (12–62)
Cigarette consumption, n (%)	
Non-smoker	14 (37.8)
Smoker	23 (62.2)
Common comorbidities, n (%)	
Hypertension	8 (21.6)
Coronary artery disease	2 (5.4)
Cardiac arrhythmia	4 (10.8)
COPD	10 (27.0)
Pneumonitis	7 (18.9)
Asthma	1 (2.7)
Diabetes mellitus	1 (2.7)
Nephritis	1 (2.7)
Other solid tumor	1 (2.7)

a Value represents median (range).

ECOG, Eastern Cooperative Oncology Group; BMI, body mass index; ALT, alanine transaminas; AST, aspartate transaminase; COPD, chronic obstructive pulmonary disease.

**Table II:** Tumor characteristics (n=37).

Characteristic	Patients, n (%)
Stage	
I	1 (2.7)
II	3 (8.1)
III	20 (54.1)
IV	13 (35.1)
Primary tumor location	
Neck	2 (5.4)
Upper chest	13 (35.1)
Middle chest	12 (32.4)
Lower chest	6 (16.2)
Not applicable	4 (10.8)
Tumor length, cm	
≤3	2 (5.4)
>3 to <5	21 (56.7)
≥5 to <7	5 (13.5)
≥7	7 (18.9)
Not applicable	2 (5.4)

**Table III:** Treatment characteristics (n=37).

Characteristic	Value
Method of treatment, n (%)	
RT alone	17 (45.9)
CRT	20 (54.1)
RT therapy technique, n (%)	
2D-RT	1 (2.7)
3D-RT	12 (31.4)
IMRT	19 (51.4)
IGRT	2 (5.4)
VMAT	3 (8.1)
Delivered RT dose, Gy	51.5 (10–66)^a^
≤50, n (%)	16 (43.2)
>50, n (%)	21 (56.8)
Discontinuation of RT, n (%)	
Yes	10 (27.0)
No	27 (73.0)
Delay of radiation, week	
≤1, n (%)	3 (8.1)
>1, n (%)	7 (18.9)
Combined chemotherapy regimen, n (%)^b^	
5-FU/cisplatin	7 (35.0)
5-FU/oxaliplatin/calcium folinate	2 (10.0)
5-FU/oxaliplatin	1 (5.0)
Cisplatin/paclitaxel	1 (5.0)
Docetaxel/cisplatin	1 (5.0)
Paclitaxel/oxaliplatin	5 (25.0)
Paclitaxel	1 (5.0)
Xeloda	1 (5.0)
Cisplatin/paclitaxel/cetuximab	1 (5.0)

a Value represents median (range);

b Data available for 20 patients.

RT, radiation; CRT, chemoradiation; 2/3D, two/three-dimensional; IMRT, intensity-modulated radiation therapy; IGRT, image guide radiation therapy; VMAT, volumetric modulated arc therapy; 5-FU, 5-fluorouracil.

**Table IV:** Acute toxicity (n=37).

	Patients, n (%)	
	
Toxicity	RT	CRT
Acute esophagitis grade		
0	8 (21.6)	14 (37.8)
1	0 (0.0)	1 (2.7)
2	8 (21.6)	3 (8.1)
3–4	1 (2.7)	2 (5.4)
Acute pneumonitis grade		
0	15 (40.5)	17 (45.9)
1	1 (2.7)	0 (0.0)
2	0 (0.0)	1 (2.7)
3–4	1 (2.7)	2 (5.4)
Radiodermatitis		
0	17 (45.9)	19 (51.3)
1	0 (0.0)	0 (0.0)
2–4	0 (0.0)	1 (2.7)
Anemia		
0	14 (37.8)	14 (37.8)
1	3 (8.1)	6 (16.2)
2–4	0 (0.0)	0 (0.0)
Acute neutropenia grade		
0	12 (32.4)	9 (24.3)
1	1 (2.7)	2 (5.4)
2	3 (8.1)	5 (13.5)
3	1 (2.7)	3 (8.1)
4	0 (0.0)	1 (2.7)
Thrombocytopenia		
0	14 (37.8)	14 (37.8)
1	3 (8.1)	2 (5.4)
2	0 (0.0)	3 (8.1)
3–4	0 (0.0)	1 (2.7)
Gastrointestinal reactions		
0	14 (37.8)	10 (27)
1	1 (2.7)	5 (13.5)
2	2 (5.4)	5 (13.5)
3–4	0 (0.0)	3 (8.1)
Any grade 3 toxicity	14 (37.8)	
Any grade 4 toxicity	1 (2.7)	

RT, radiation; CRT, chemoradiation.

**Table V:** Comparison of patient age and outcome in specific CRT clinical trials of elderly patients with EC.

Authors (ref)	n	Median age, years	Median OS, months	Median PFS, months	ORR, %	Any grade 3–4 toxicity, %	Grade 3–4 hemotoxicity, %
Current study	20	76	17	14	65.7	40.5	13.5
Mak *et al*(15)	34	79.5	12	10.4	NR	73.5	35.2
Servagi-Vernat *et al*(16)	22	79.4	15	11.2	63.3	NR	13
Anderson S *et al*(19)	23	77	35	NR	68	36	36
Tougeron *et al*(20)	282	76.5	9.7	NR	NR	17	NR
Tougeron *et al*(21)	109	74.4	15.2	8.3	57.8	25.6	19
Go *et al*(22)	57	69	11.2	NR	84.4	73.7	18.4

CRT; chemoradiation; EC, esophageal cancer; OS, overall survival; PFS, progression-free survival; ORR, objective remission rate; NR, not reported.

## References

[1] National Cancer Institute Cancer Statistics: SEER stat fact sheets: esophagus.

[2] Zhang SW, Lei ZL, Li GL, Zou XL, Zhao P, Chen WQ (2010). A report of cancer incidence and mortality from 34 cancer registries in China, 2006. China Cancer.

[3] Gholipour C, Shalchi RA, Abbasi M (2008). A histopathological study of esophageal cancer on the western side of the Caspian littoral from 1994 to 2003. Dis Esophagus.

[4] Tran GD, Sun XD, Abnet CC (2005). Prospective study of risk factors for esophageal and gastric cancers in the Linxian general population trial cohort in China. Int J Cancer.

[5] Sun XD, Yu JM, Fan XL, Ren RM, Li MH, Zhang GL (2006). Randomized clinical study of surgery versus radiotherapy alone in the treatment of resectable esophageal cancer in the chest. Zhonghua Zhong Liu Za Zhi.

[6] Abrams JA, Buono DL, Strauss J, McBride RB, Hershman DL, Neugut AI (2009). Esophagectomy compared with chemoradiation for early stage esophageal cancer in the elderly. Cancer.

[7] Smith TJ, Ryan LM, Douglass HO (1998). Combined chemoradiotherapy vs. radiotherapy alone for early stage squamous cell carcinoma of the esophagus: a study of the Eastern Cooperative Oncology Group. Int J Radiat Oncol Biol Phys.

[8] Wobbes T, Baron B, Paillot B (2001). Prospective randomised study of split-course radiotherapy versus cisplatin plus split-course radiotherapy in inoperable squamous cell carcinoma of the oesophagus. Eur J Cancer.

[9] National Comprehensive Cancer Network (2012). Clinical Practice Guidelines in Oncology™ version 2.

[10] Freeman RK, Ascioti AJ, Mahidhara RJ (2012). Palliative therapy for patients with unresectable esophageal carcinoma. Surg Clin North Am.

[11] Ikeda E, Kojima T, Kaneko K (2011). Efficacy of concurrent chemoradiotherapy as a palliative treatment in stage IVB esophageal cancer patients with dysphasia. Jpn J Clin Oncol.

[12] Law S, Wong KH, Kwok KF, Chu KM, Wong J (2004). Predictive factors for postoperative pulmonary complications and mortality after esophagectomy for cancer. Ann Surg.

[13] Nishino M, Jackman DM, Hatabu H (2010). New Response Evaluation Criteria in Solid Tumours (RECIST) guidelines for advanced non-small cell lung cancer: comparison with original RECIST and impact on assessment of tumor response to targeted therapy. AJR Am J Roentgenol.

[14] National Cancer Institute Cancer Therapy Evaluation Program: protocol development.

[15] Mak HK, Mamon HJ, Ryan DP (2010). Toxicity and outcomes after chemoradiation for esophageal cancer in patients age 75 or older. Dis Esophagus.

[16] Servagi-Vernat S, Bosset M, Crehange G (2009). Feasibility of chemoradiotherapy for oesophageal cancer in elderly patients aged >or=75 years: a prospective, single-arm phase II study. Drug Aging.

[17] Herskovic A, Martz K, al-Sarraf M (1992). Combined chemotherapy and radiotherapy compared with radiotherapy alone in patients with cancer of the esophagus. N Engl J Med.

[18] Cooper JS, Guo MD, Herskovic A, Radiation Therapy Oncology Group (1999). Chemoradiotherapy of locally advanced esophageal cancer: long-term follow-up of a prospective randomized trial (RTOG 85-01). JAMA.

[19] Anderson SE, Minsky BD, Bains M, Hummer A, Kelsen D, Ilson DH (2007). Combined modality chemoradiation in elderly oesophageal cancer patients. Br J Cancer.

[20] Tougeron D, Hamidou H, Scotté M, Di Fiore F, Antonietti M, Paillot B, Michel P (2010). Esophageal cancer in the elderly: an analysis of the factors associated with treatment decisions and outcomes. BMC Cancer.

[21] Tougeron D, Di Fiore F, Thureau S (2008). Safety and outcome of definitive chemoradiaotherapy in elderly patients with oesophageal cancer. Br J Cancer.

[22] Go SI, Sup Lee W, Hee Kang M (2012). Response to concurrent chemoradiotherapy as a prognostic marker in elderly patients with locally advanced esophageal cancer. Tumori.

[23] Coia LR, Minsky BD, Berkey BA (2000). Outcome of patients receiving radiation for cancer of the esophagus: results of the 1992–1994 Patterns of Care Study. J Clin Oncol.

[24] Polee MB, Hop WC, Kok TC (2003). Prognostic factors for survival in patients with advanced oesophageal cancer treated with cisplatin-based combination chemotherapy. Br J Cancer.

[25] Rohatgi P, Swisher SG, Correa AM (2005). Characterization of pathologic complete response after preoperative chemoradiotherapy in carcinoma of the esophagus and outcome after pathologic complete response. Cancer.

[26] Di Fiore F, Lecleire S, Rigal O (2006). Predictive factors of survival in patients treated with definitive chemoradiotherapy for squamous cell esophageal carcinoma. World J Gastroenterol.

[27] Takeuchi S, Ohtsu A, Doi T (2007). A retrospective study of definitive chemoradiotherapy for elderly patients with esophageal cancer. Am J Clin Oncol.

